# Supplementary material for: Novel Thienopyrimidine-Hydrazinyl Compounds Induce DRP1-Mediated Non-Apoptotic Cell Death in Triple-Negative Breast Cancer Cells
Source: Cancers (Basel). 2024 Jul 23;16(15):2621. doi: 10.3390/cancers16152621 (PMC11311031; doi:10.3390/cancers16152621)
Supplement: Supplementary file 1 [file cancers-16-02621-s001.zip › Original Western Blot Images.pdf]

Figure 3c

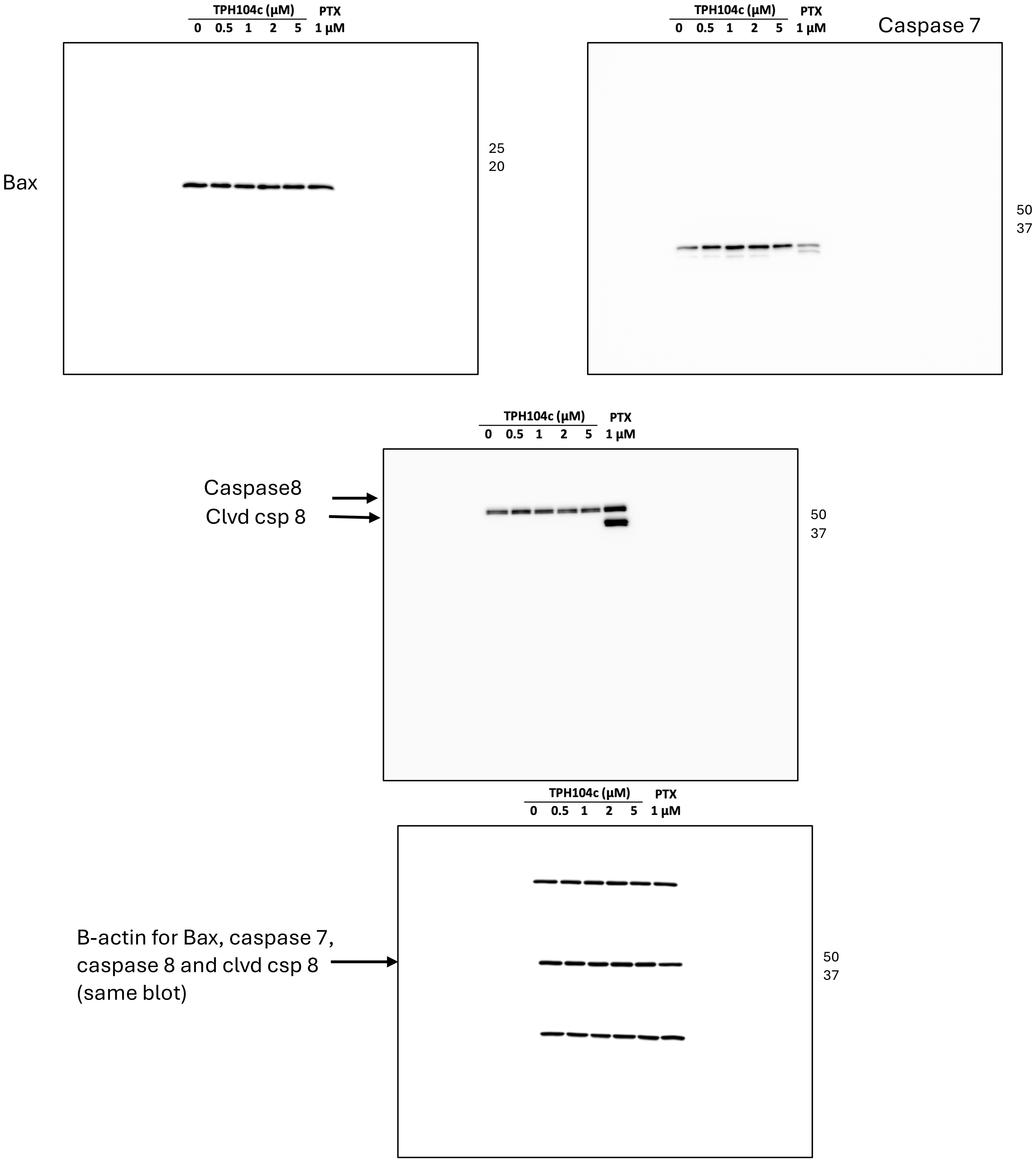

TPH104c (μM)
0 0.5 1 2 5
PTX
1 μM
Bax
25
20
Caspase 7
50
37
Caspase8
Clvd csp 8
B-actin for Bax, caspase 7, caspase 8 and clvd csp 8 (same blot)

Figure 3c

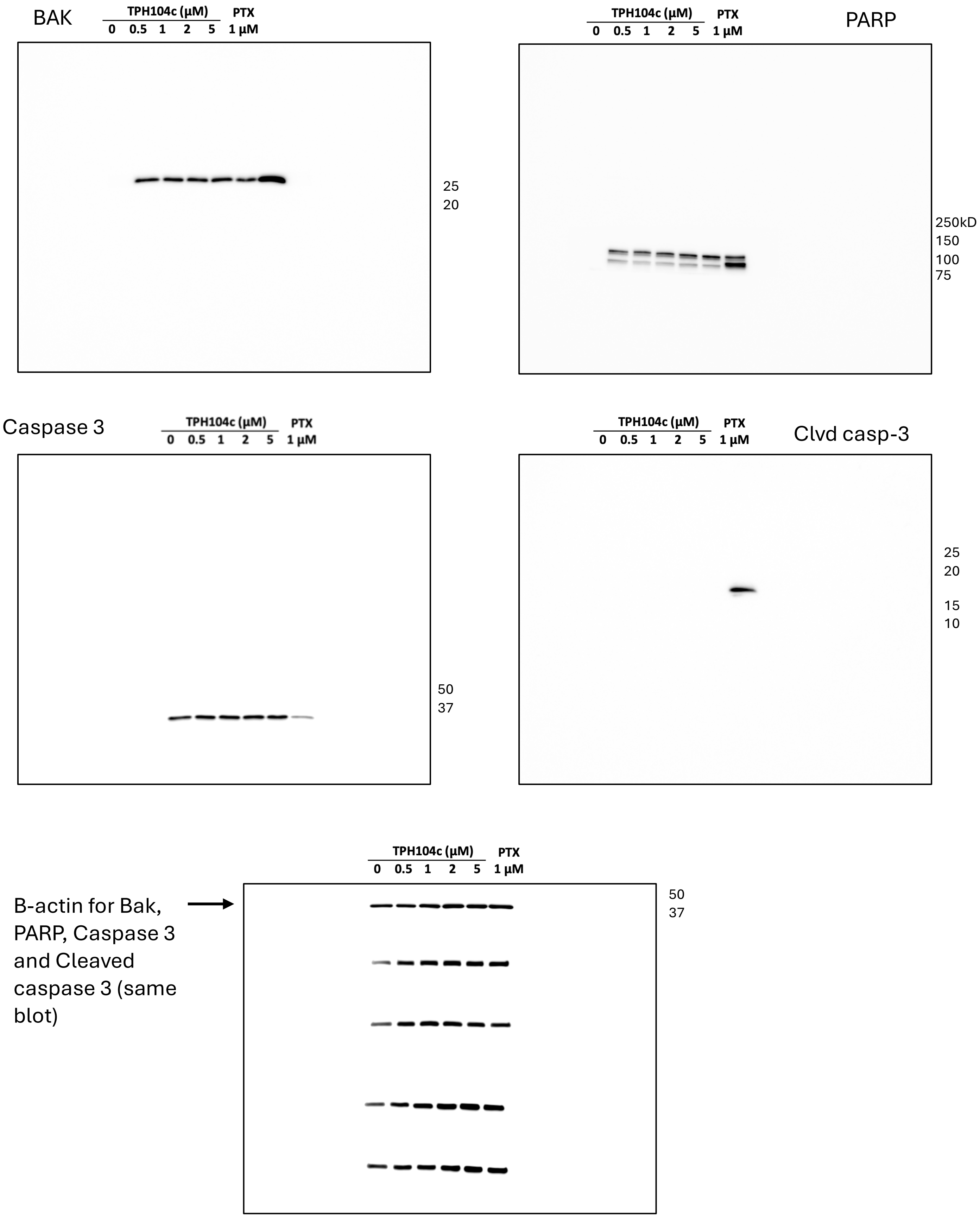
BAK
TPH104c (μM)
0 0.5 1 2 5
PTX 1 μM
25
20
PARP
TPH104c (μM)
0 0.5 1 2 5
PTX 1 μM
250kD
150
100
75
Caspase 3
TPH104c (μM)
0 0.5 1 2 5
PTX 1 μM
50
37
Clvd casp-3
TPH104c (μM)
0 0.5 1 2 5
PTX 1 μM
25
20
15
10
B-actin for Bak, PARP, Caspase 3 and Cleaved caspase 3 (same blot)
TPH104c (μM)
0 0.5 1 2 5
PTX 1 μM
50
37

Figure 3c

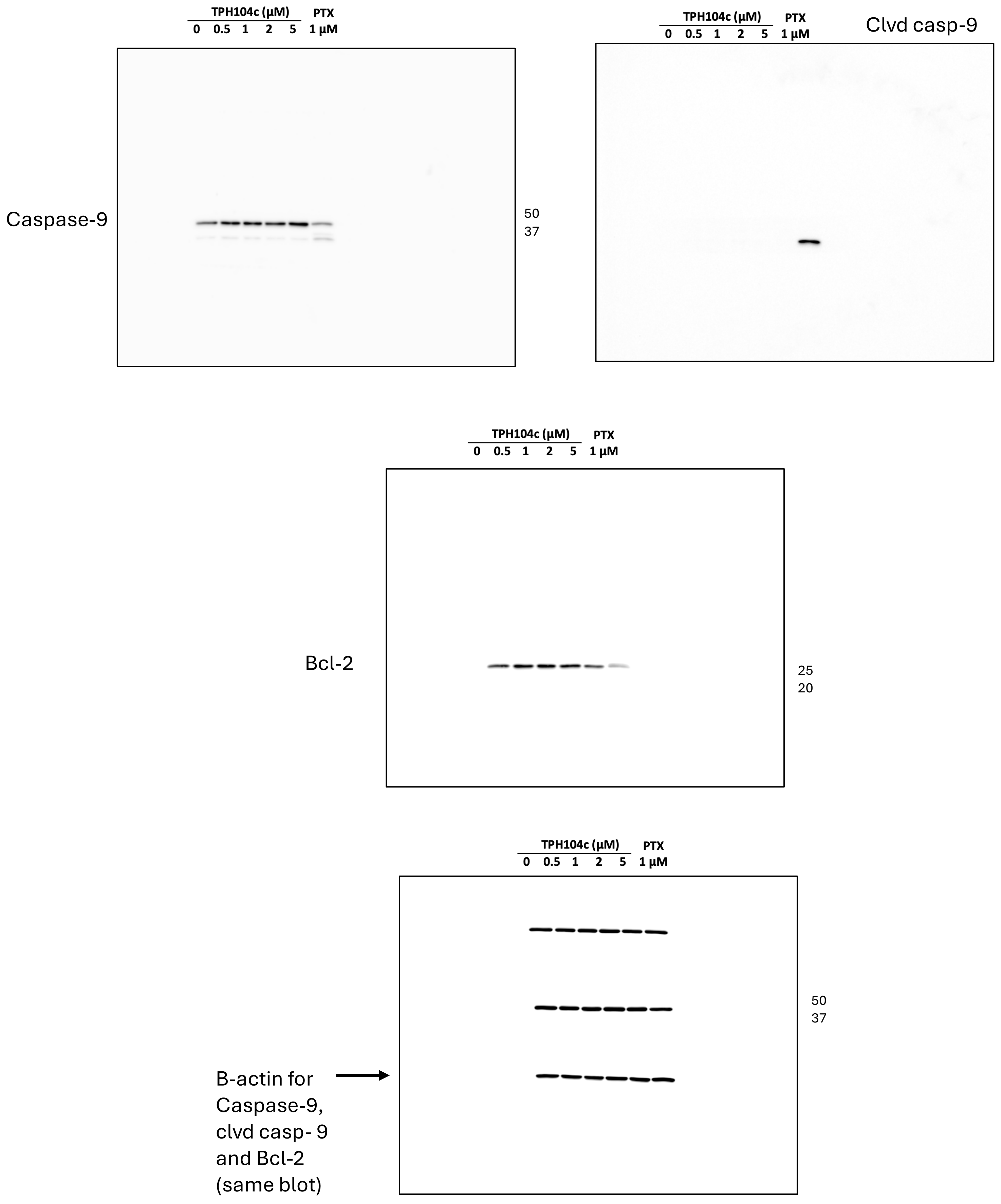
TPH104c (μM)
PTX
0 0.5 1 2 5 1 μM
Caspase-9
50
37
TPH104c (μM)
PTX
0 0.5 1 2 5 1 μM
Clvd casp-9
50
37
TPH104c (μM)
PTX
0 0.5 1 2 5 1 μM
Bcl-2
25
20
TPH104c (μM)
PTX
0 0.5 1 2 5 1 μM
50
37
B-actin for Caspase-9, clvd casp- 9 and Bcl-2 (same blot)

Figure 3c

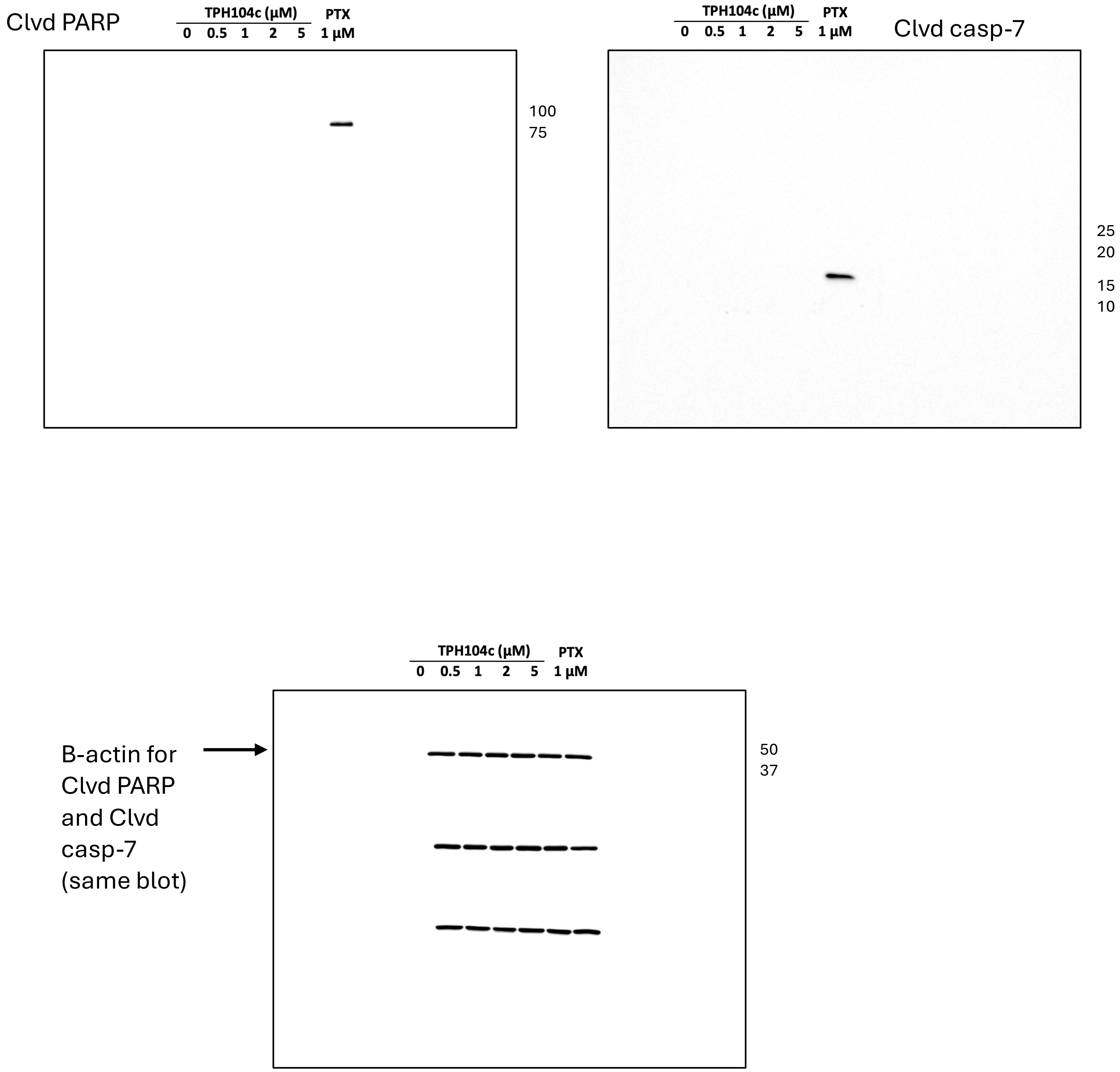
Clvd PARP
TPH104c (μM)
0 0.5 1 2 5
PTX
1 μM
100
75
TPH104c (μM)
0 0.5 1 2 5
PTX
1 μM
Clvd casp-7
25
20
15
10
TPH104c (μM)
0 0.5 1 2 5
PTX
1 μM
B-actin for Clvd PARP and Clvd casp-7 (same blot)
50
37

Figure 4c

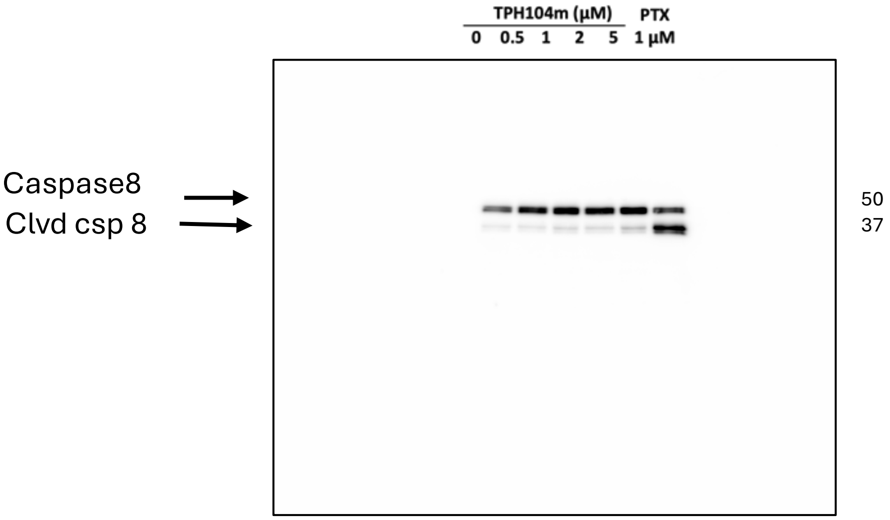
TPH104m (μM)
PTX
0 0.5 1 2 5 1 μM
Caspase8
Clvd csp 8
50
37

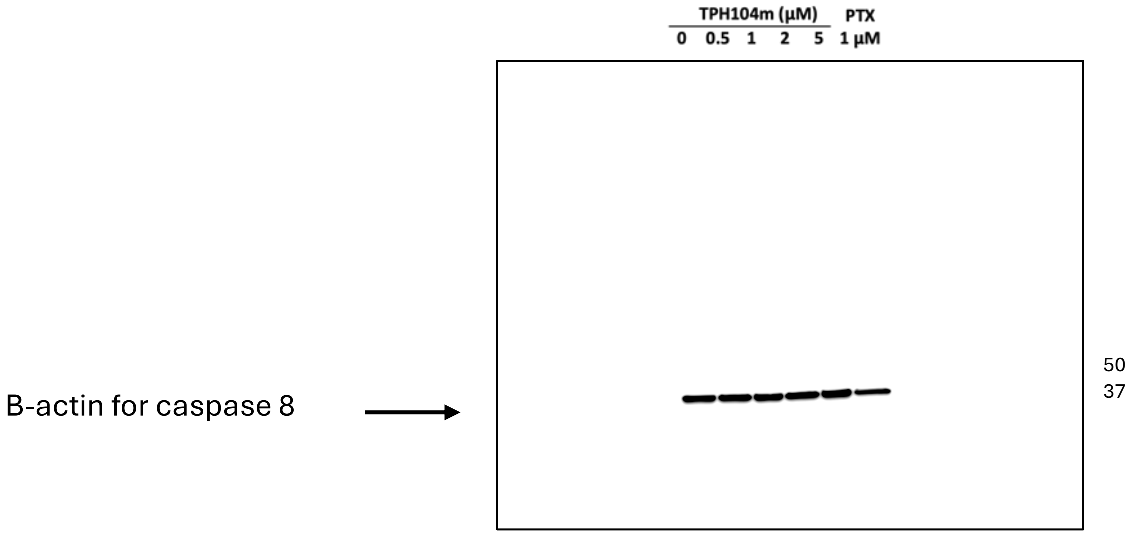
TPH104m (μM)
PTX
0 0.5 1 2 5 1 μM
B-actin for caspase 8
50
37

Figure 4c

PARP

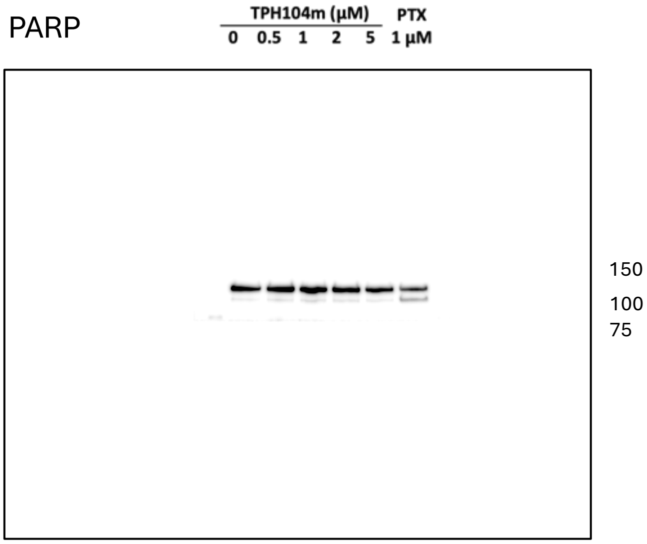

Clvd PARP

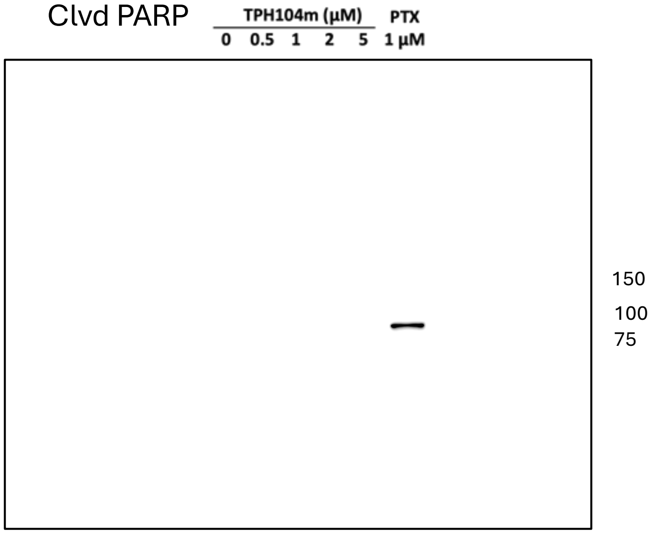

B-actin for Clvd PARP and PARP (same blot)

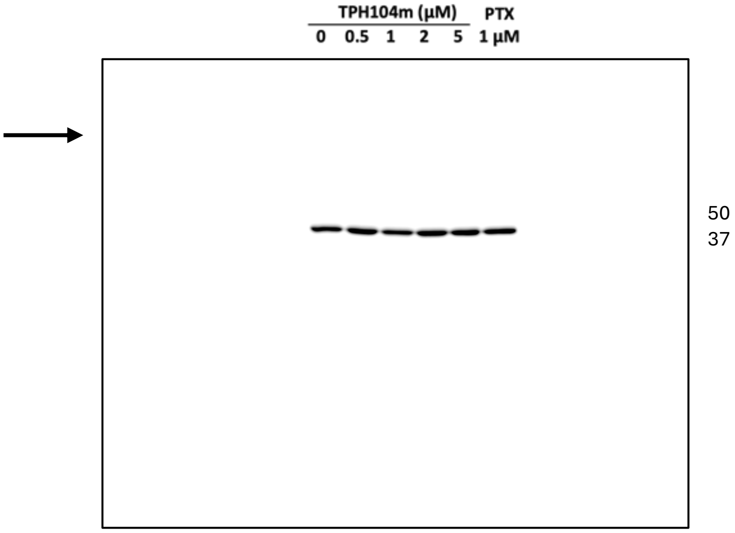

Figure 4c

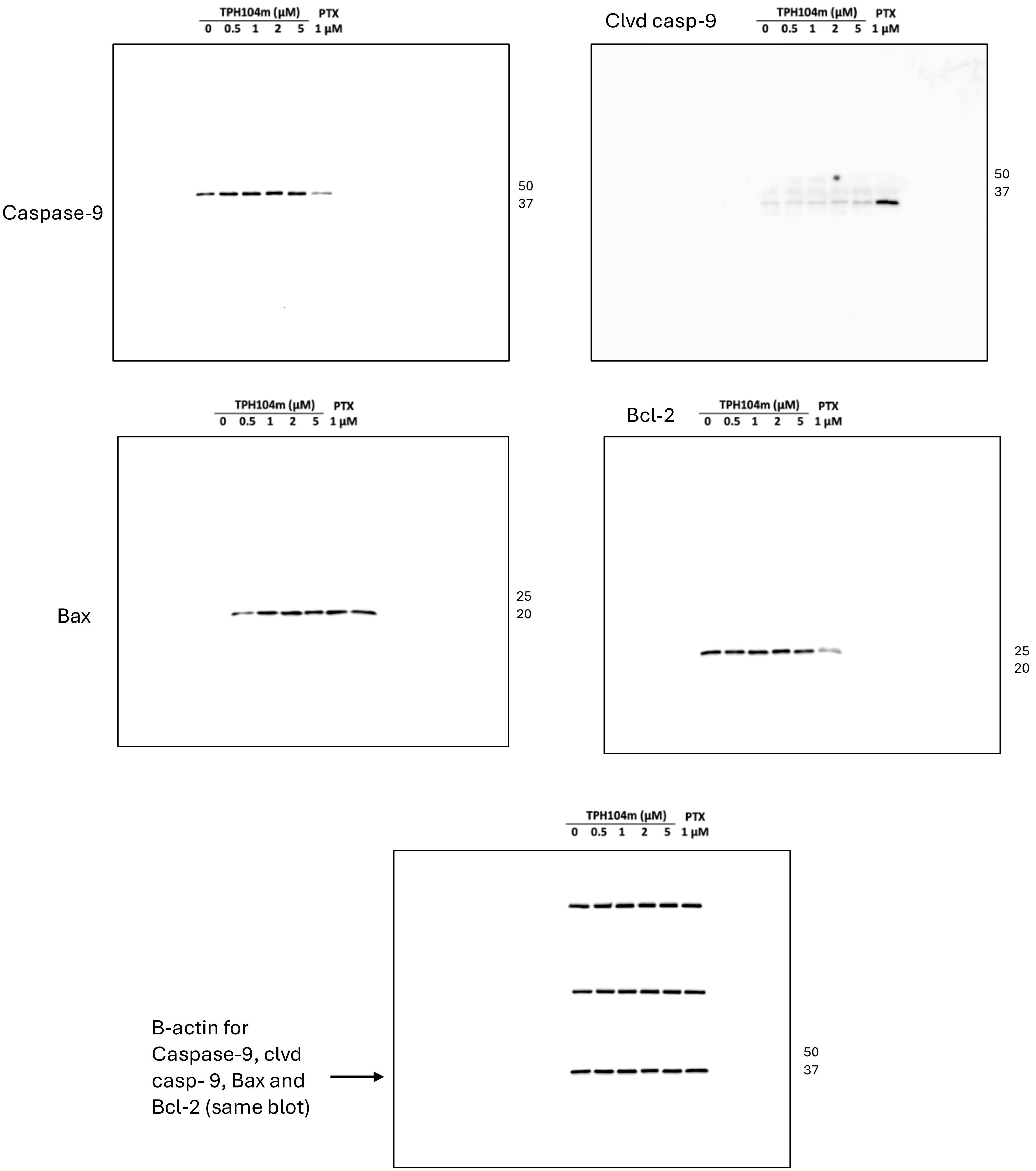

TPH104m (µM) PTX
0 0.5 1 2 5 1 µM
Caspase-9
50
37
Clvd casp-9
TPH104m (µM) PTX
0 0.5 1 2 5 1 µM
50
37
TPH104m (µM) PTX
0 0.5 1 2 5 1 µM
Bax
25
20
Bcl-2
TPH104m (µM) PTX
0 0.5 1 2 5 1 µM
25
20
TPH104m (µM) PTX
0 0.5 1 2 5 1 µM
B-actin for Caspase-9, clvd casp- 9, Bax and Bcl-2 (same blot)
50
37

Figure 4c

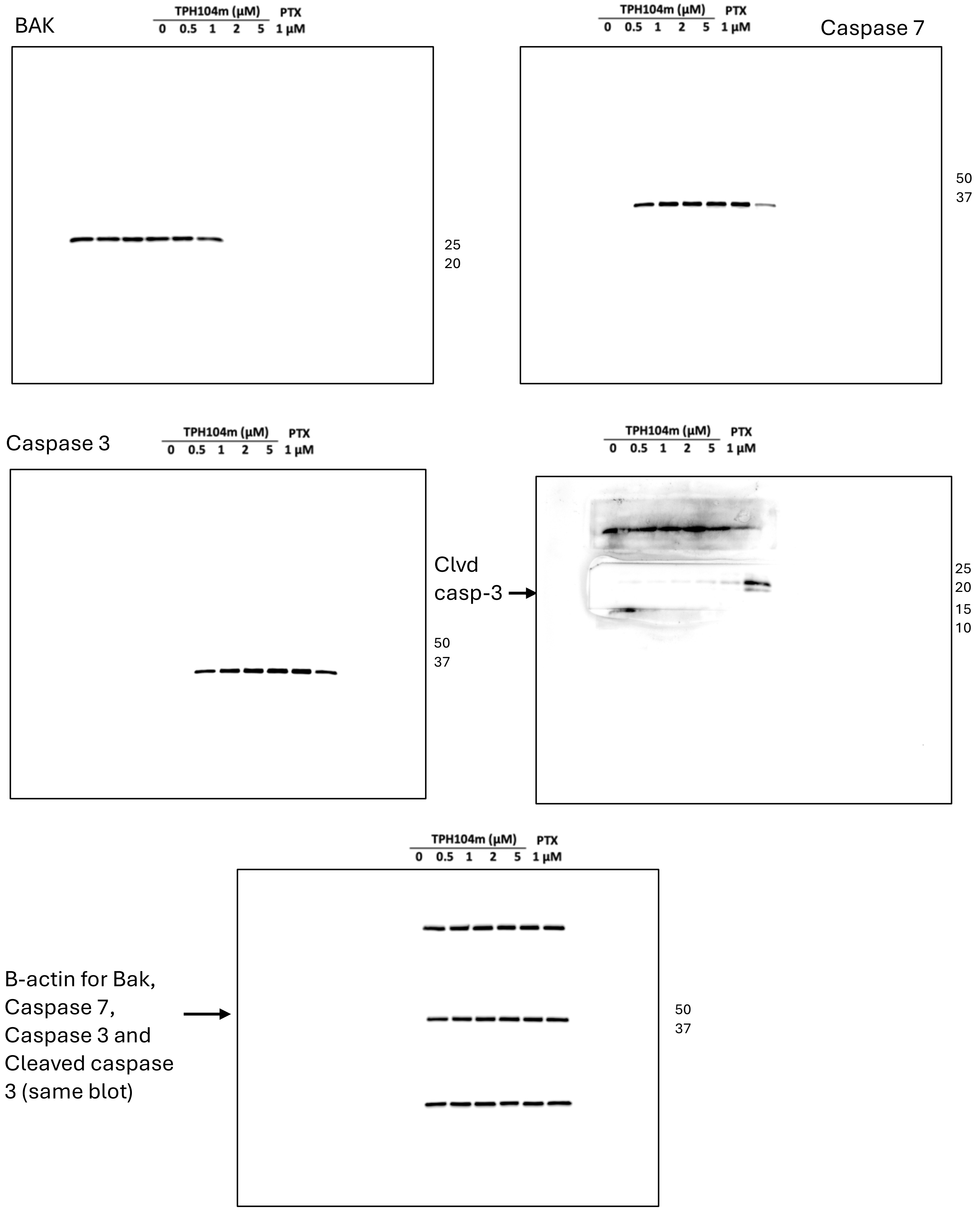
BAK
TPH104m (µM) PTX
0 0.5 1 2 5 1 µM
25
20
Caspase 7
TPH104m (µM) PTX
0 0.5 1 2 5 1 µM
50
37
Caspase 3
TPH104m (µM) PTX
0 0.5 1 2 5 1 µM
50
37
Clvd casp-3
TPH104m (µM) PTX
0 0.5 1 2 5 1 µM
25
20
15
10
TPH104m (µM) PTX
0 0.5 1 2 5 1 µM
B-actin for Bak, Caspase 7, Caspase 3 and Cleaved caspase 3 (same blot)
50
37

Figure 4c

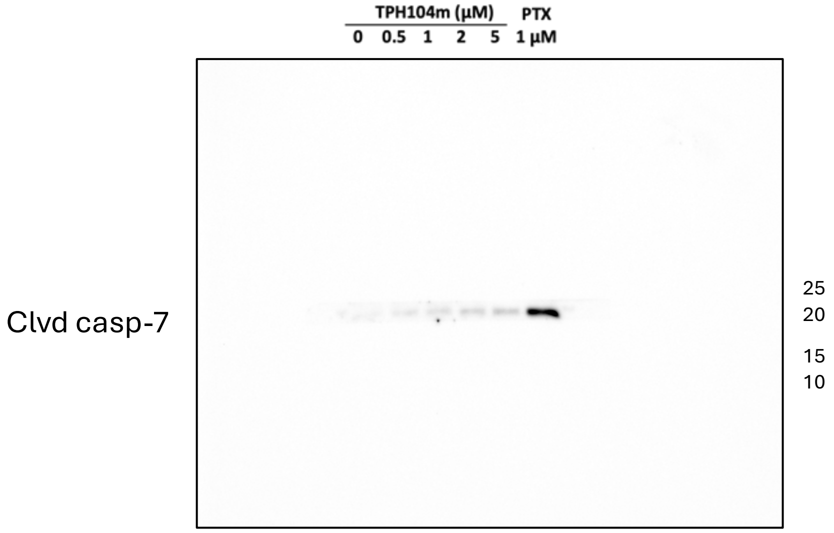
TPH104m (µM)
0 0.5 1 2 5
PTX
1 µM
Clvd casp-7
25
20
15
10

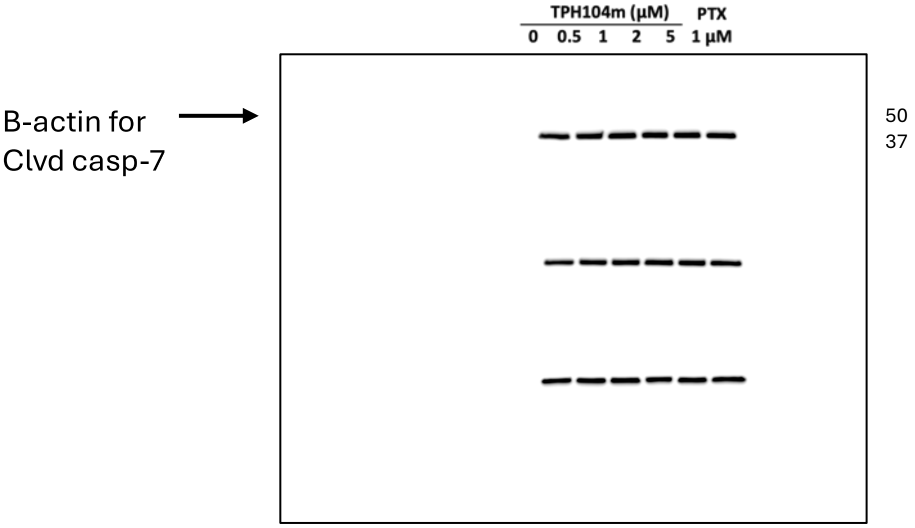
TPH104m (µM)
0 0.5 1 2 5
PTX
1 µM
B-actin for
Clvd casp-7
50
37

Figure 6a

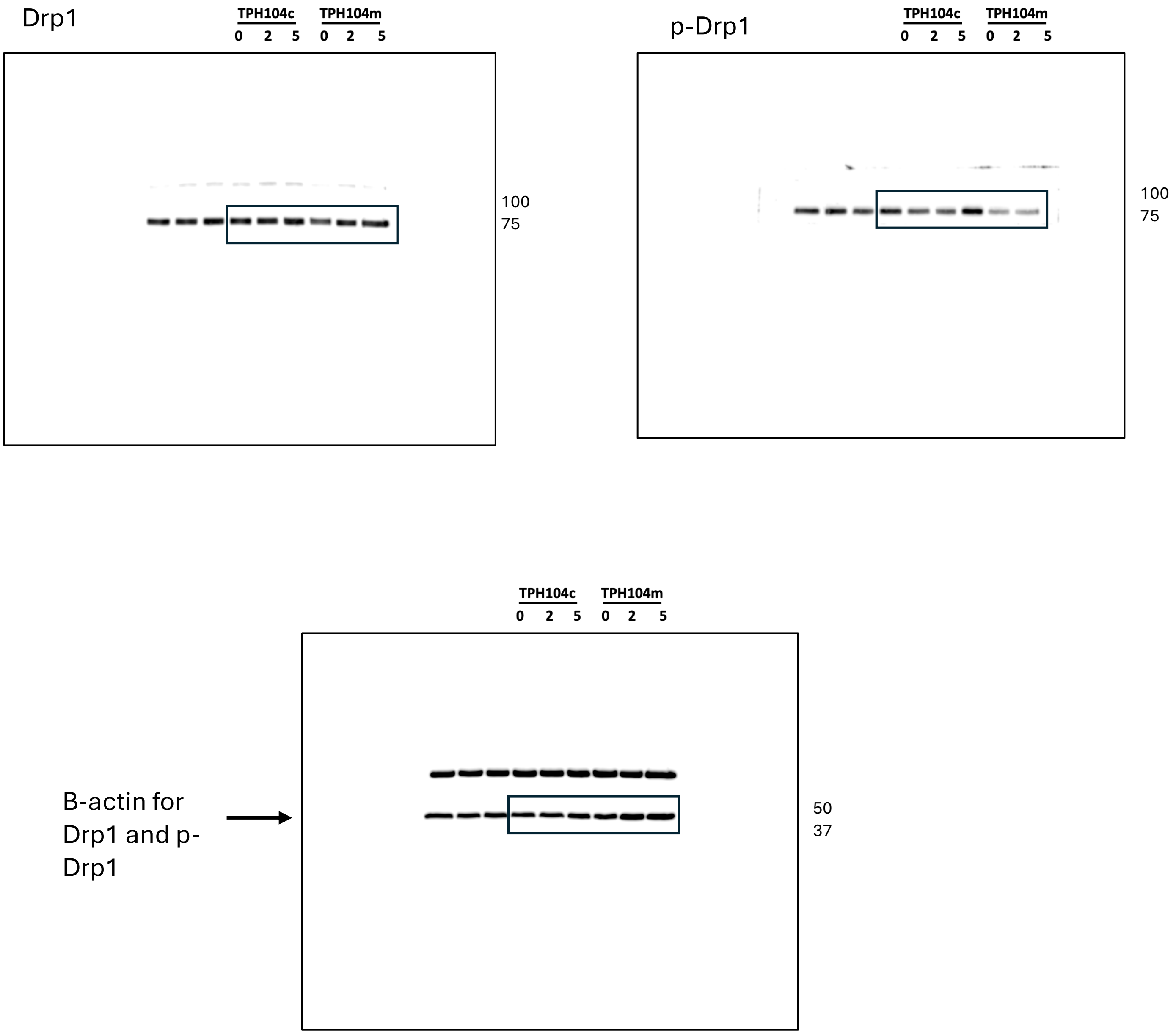
Drp1
TPH104c
TPH104m
0 2 5 0 2 5
100
75
p-Drp1
TPH104c
TPH104m
0 2 5 0 2 5
100
75
TPH104c
TPH104m
0 2 5 0 2 5
B-actin for Drp1 and p-Drp1
50
37

Figure 6a

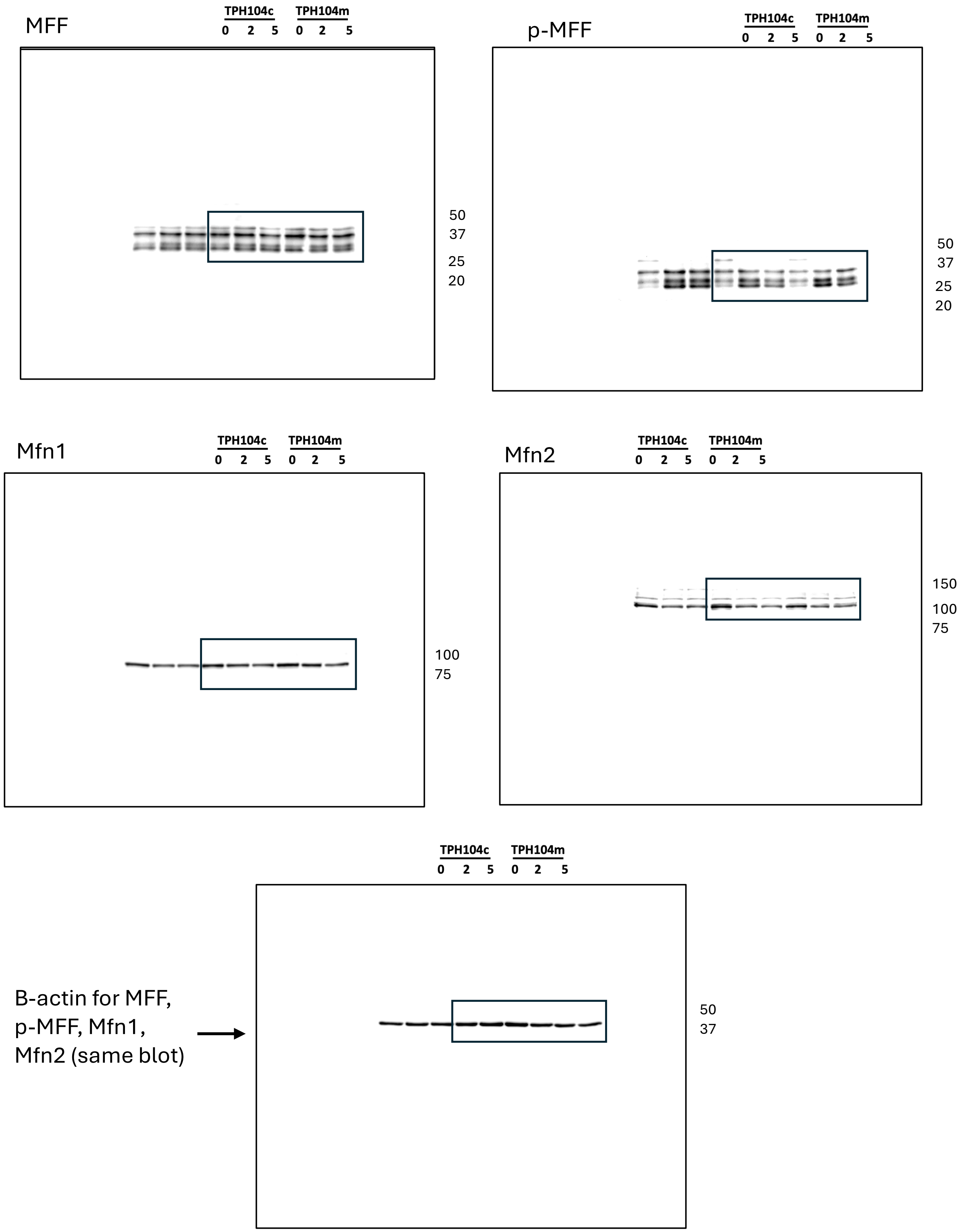
MFF
TPH104c
0 2 5
TPH104m
0 2 5
50
37
25
20
p-MFF
TPH104c
0 2 5
TPH104m
0 2 5
50
37
25
20
Mfn1
TPH104c
0 2 5
TPH104m
0 2 5
100
75
Mfn2
TPH104c
0 2 5
TPH104m
0 2 5
150
100
75
TPH104c
0 2 5
TPH104m
0 2 5
B-actin for MFF, p-MFF, Mfn1, Mfn2 (same blot)
50
37

Figure 6a

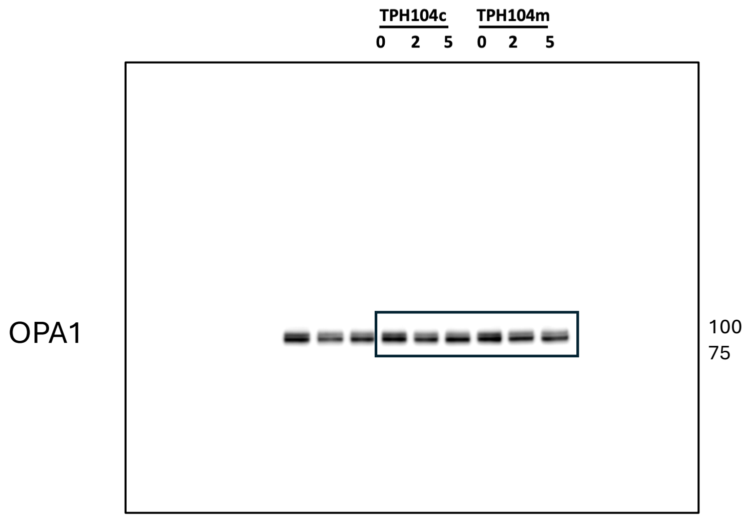
TPH104c
TPH104m
0 2 5 0 2 5
OPA1
100
75

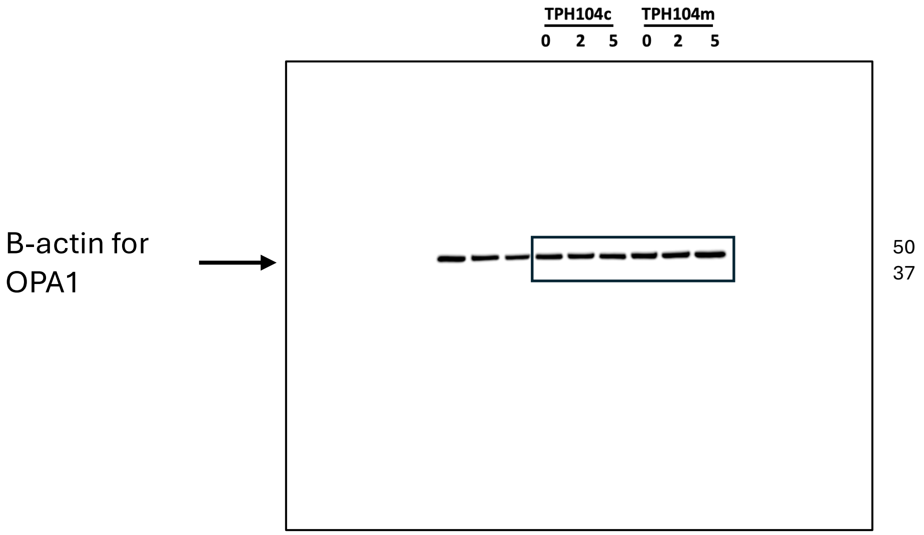
TPH104c
TPH104m
0 2 5 0 2 5
B-actin for OPA1
50
37

Figure 6a

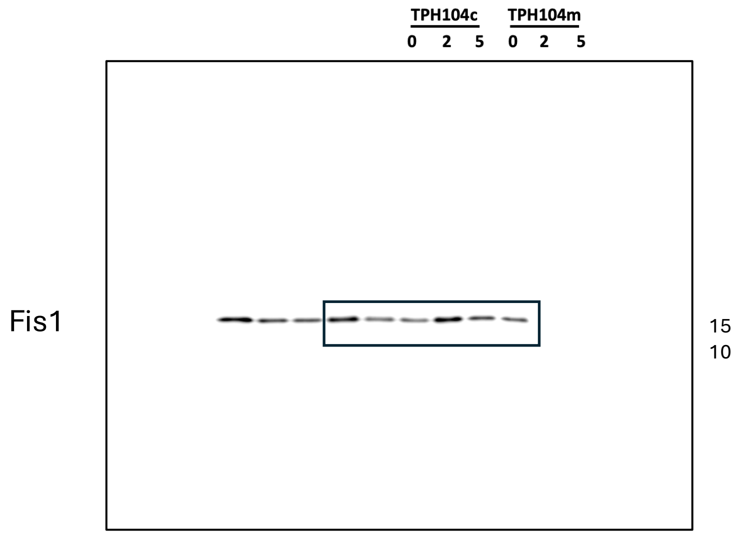
TPH104c
TPH104m
0 2 5 0 2 5
Fis1
15
10

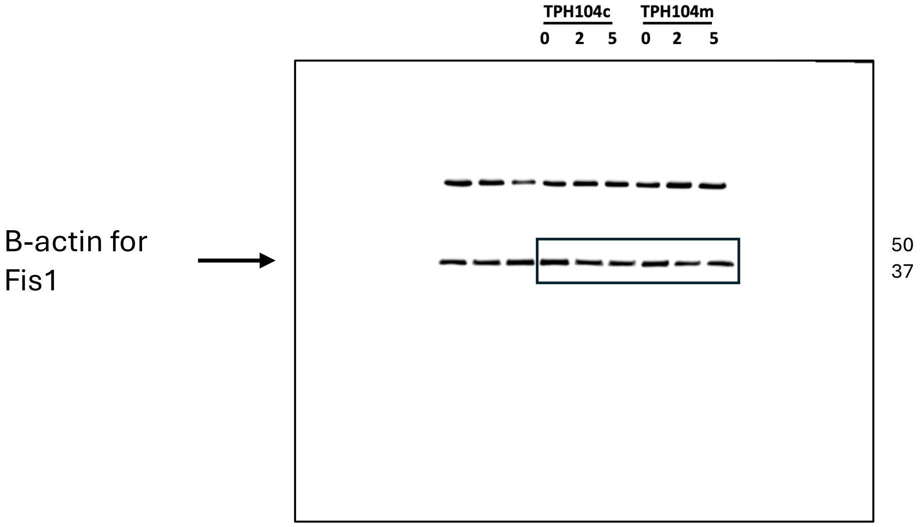
TPH104c
TPH104m
0 2 5 0 2 5
B-actin for Fis1
50
37

Figure 7a

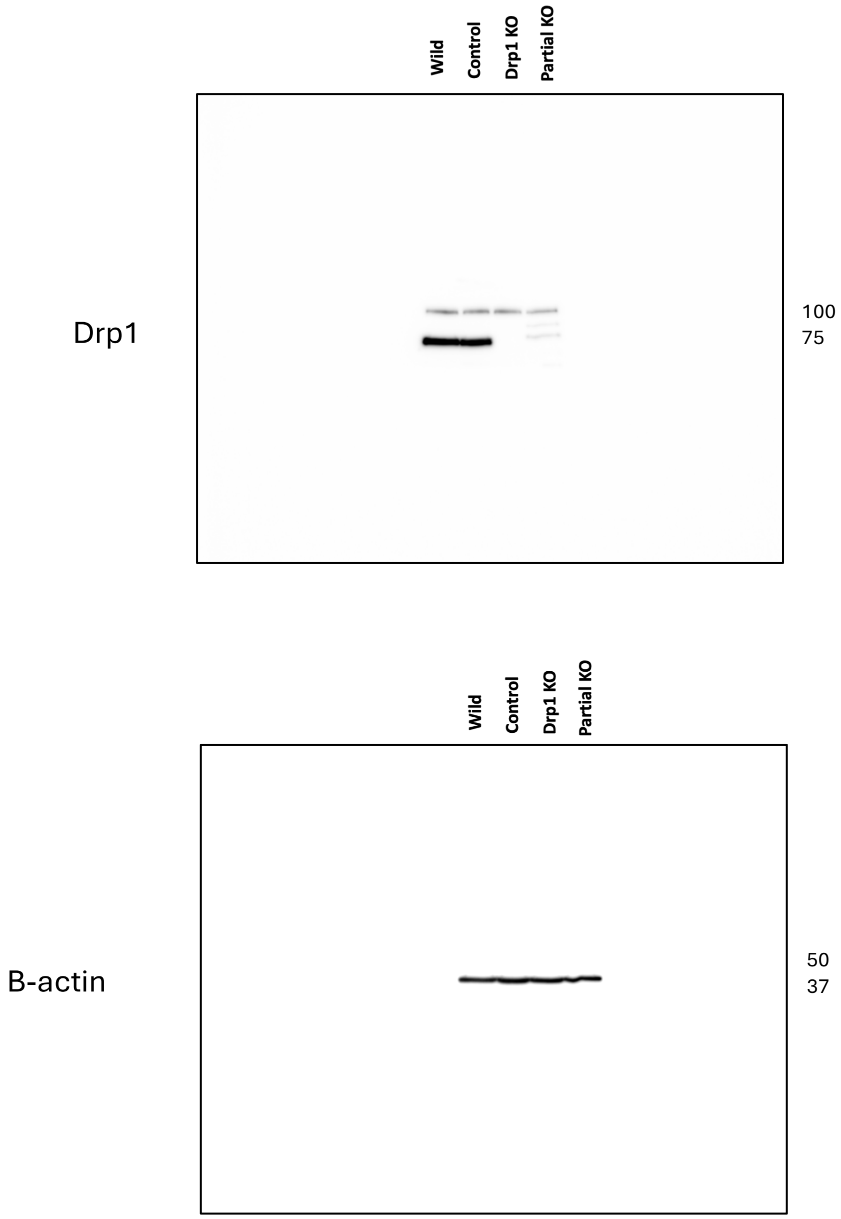
Wild
Control
Drp1 KO
Partial KO
Drp1
100
75
Wild
Control
Drp1 KO
Partial KO
B-actin
50
37
