# Supplementary material for: Novel Thienopyrimidine-Hydrazinyl Compounds Induce DRP1-Mediated Non-Apoptotic Cell Death in Triple-Negative Breast Cancer Cells
Source: Cancers (Basel). 2024 Jul 23;16(15):2621. doi: 10.3390/cancers16152621 (PMC11311031; doi:10.3390/cancers16152621)
Supplement: Supplementary file 1 [file cancers-16-02621-s001.zip › supplementary figures and table.pdf]

Supplementary Materials: Novel Thienopyrimidine-Hydrazinyl Compounds Induce DRP1-Mediated Non-Apoptotic Cell Death in Triple-Negative Breast Cancer Cells

Saloni Malla, Angelique Nyinawabera, Rabin Neupane, Rajiv Pathak, Donghyun Lee, Mariam Abou-Dahech, Shikha Kumari, Suman Sinha, Yuan Tang, Aniruddha Ray, Charles R. Ashby, Jr., Mary Qu Yang, R. Jayachandra Babu and Amit K. Tiwari

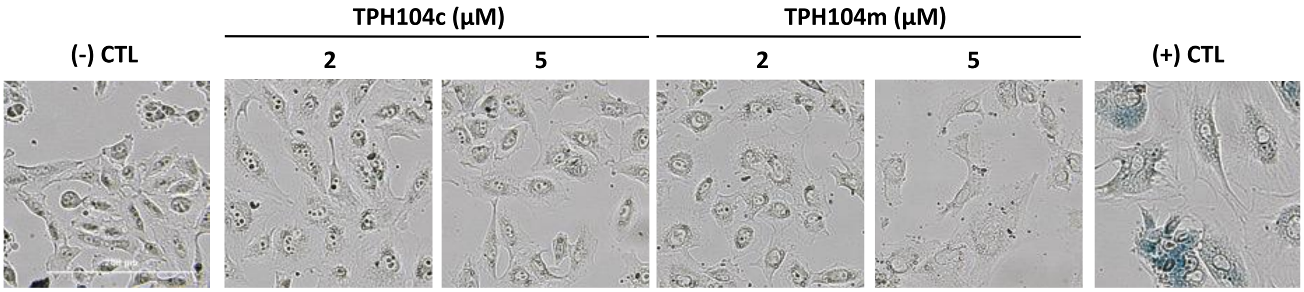

Figure S1. TPH104c and TPH104m do not induce senescence in BT-20 cells. Morphological images of MEF cells treated with 250 nM of Doxorubicin for 24 hours and BT-20 cells treated with 0.1, 0.3 or 1 µM of TPH104c and TPH104m for 24 hours and stained with β-galactosidase. Scale bar, 200 µm.

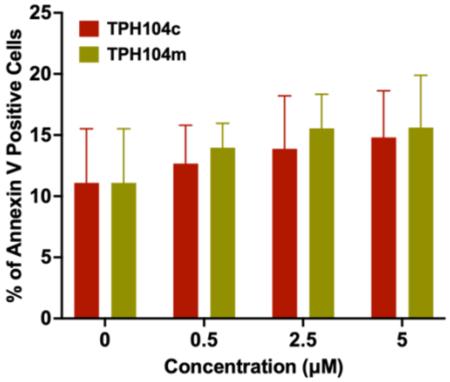

Figure S2. The effect of TPH104c and TPH104m on phosphatidylserine exposure of BT-20 cells. Bar graph illustrating percentage of Annexin V positive BT-20 cells treated with TPH104c and TPH104m or vehicle that correlates to phosphatidyl serine exposed on the surface of apoptotic cells.

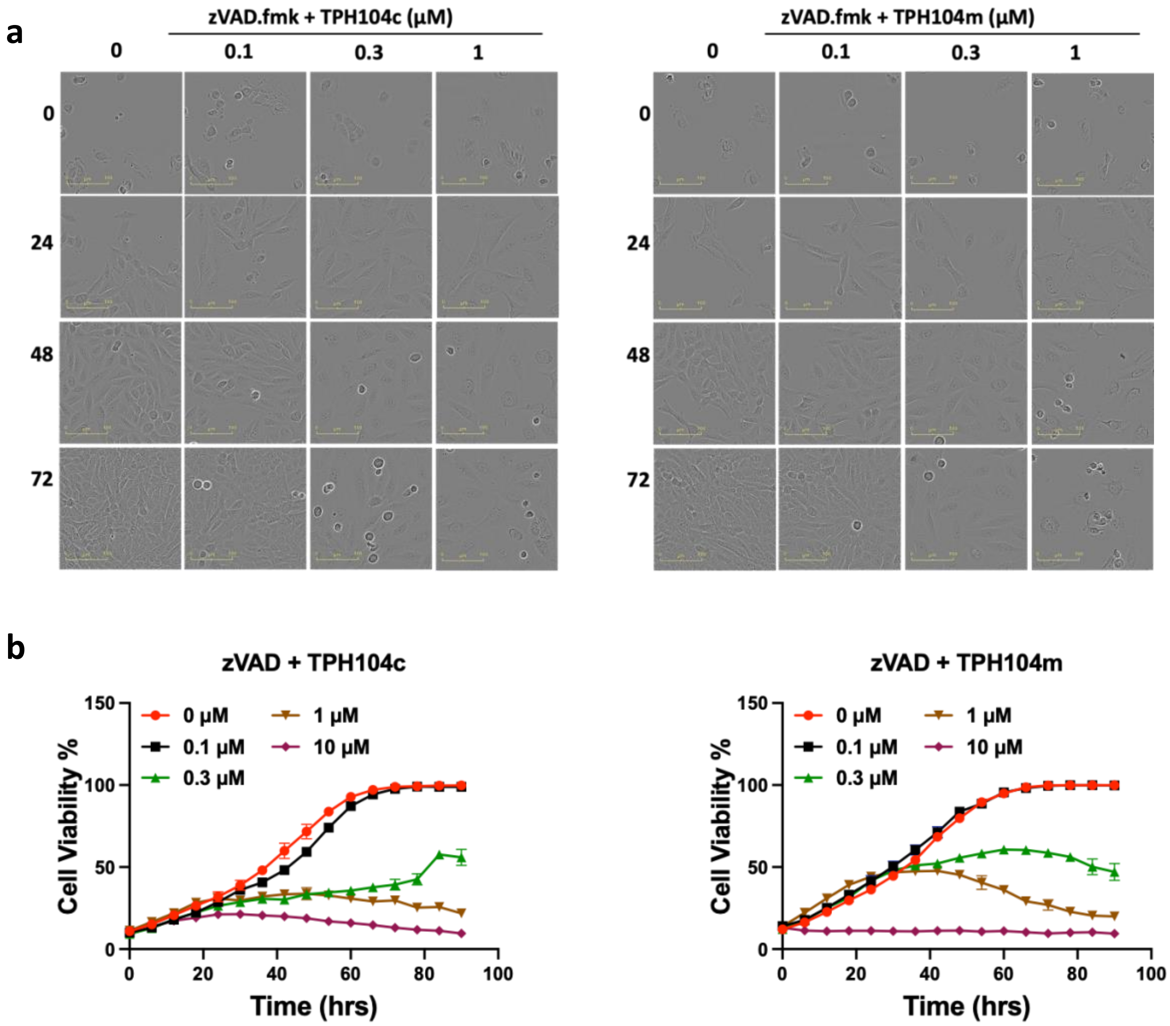

Figure S3. The impact of pan-caspase inhibitor, z-vad.fmk, TPH104c and TPH104m on caspase activity. **(a)** Morphological images of BT-20 cells pre-treated with 100 μM of zVAD.fmk and treated with 0.1, 0.3 or 1 μM of TPH104c and TPH104m captured at 0, 24, 48 and 72 hours. Scale bar, 100 μm. **(b)** The cell viability curves of BT-20 cells pre-treated with zVAD.fmk and then incubated with varying concentrations of TPH104c or TPH104m over 72 hours obtained using IncuCyte S3 software based on phase-contrast images of BT-20 cells. The data represent the mean ± SD of three independent experiments performed in triplicate.

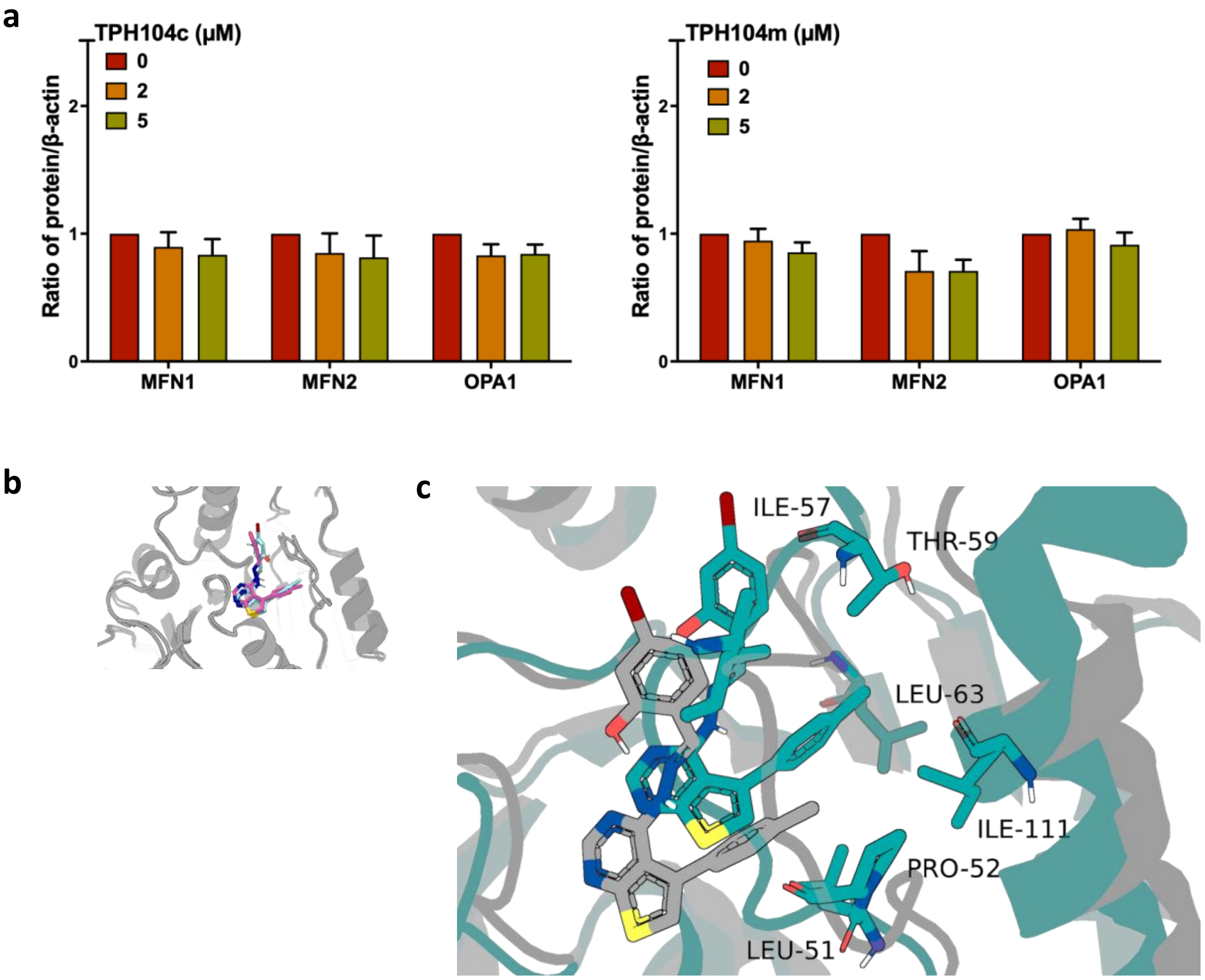

d

Comp.	PAC200 (IC_{50} (μM) ± SD)					
	Cell Titre Glo Assay			SRB Assay		
	Control	Drp1 KO	Partial Drp1 KO	Control	Drp1 KO	Partial Drp1 KO
TPH104c	6.91 ± 2.01	18.60 ± 6.71	13.03 ± 7.85	3.38 ± 1.70	9.96 ± 3.32	5.79 ± 2.19
TPH104m	2.41 ± 1.93	11.41 ± 4.75	7.60 ± 2.88	0.95 ± 0.32	6.87 ± 2.08	5.56 ± 1.66

Comp.	PAC200 (IC_{50} (μM) ± SD)					
	Cell Titre Glo Assay			SRB Assay		
	Control	Drp1 KO	Partial Drp1 KO	Control	Drp1 KO	Partial Drp1 KO
TPH104c	6.91 ± 2.01	18.60 ± 6.71	13.03 ± 7.85	3.38 ± 1.70	9.96 ± 3.32	5.79 ± 2.19
TPH104m	2.41 ± 1.93	11.41 ± 4.75	7.60 ± 2.88	0.95 ± 0.32	6.87 ± 2.08	5.56 ± 1.66

Figure S4. The effect of mitochondrial fusion proteins on TPH104c and TPH104m-induced cell death. **(a)** Histograms summarizing the ratio of fusion proteins (MFN1, MFN2 and OPA1) to β-actin and normalized to vehicle control. The data are presented as the means ± SEM of three separate studies. Predicted non-covalent interactions of the ligands TPH104c and TPH104m. **(b)** TPH104c-magenta; TPH104m-cyan **(c)** TPH104m migration to a deeper hydrophobic pocket; ligand initial pose-gray; ligand final pose-cyan; The aryl moiety was observed to delve deeper into the hydrophobic sub pocket guarded by LEU51, PRO52, ILE57, ILE111, THR59 and ILE63) **(d)** IC_{50} values of TPH104c and TPH104m in control wild-type, partial Drp1 KO and complete Drp1 KO PAC200 cells by the means of MTT, Cell Titre Blue assay, Cell Titre Blue assay and SRB assay after 72h post treatment. Data are mean ± SEM of four independent experiments, each performed in triplicate.

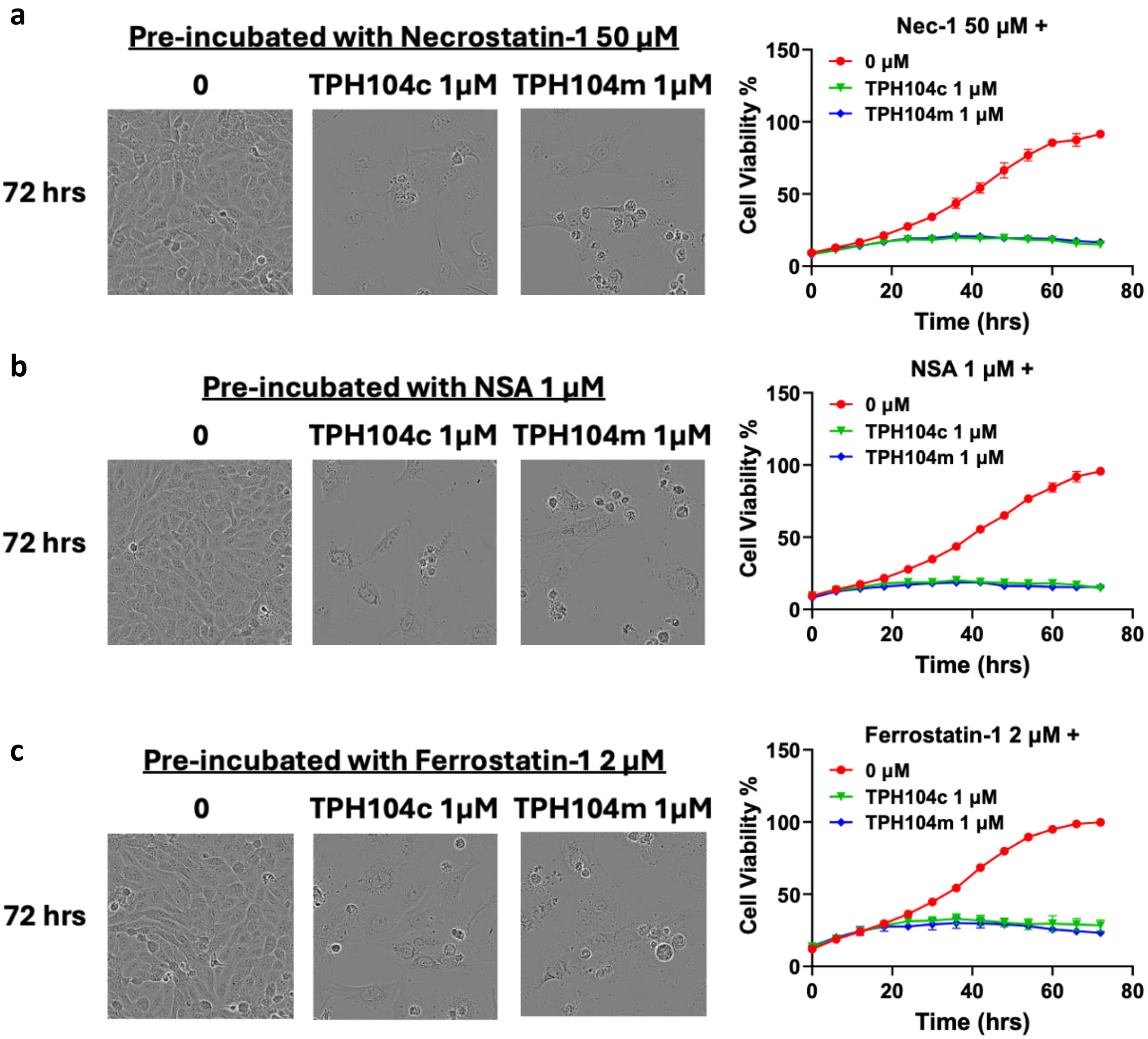

Figure S5. The impact of Necrostatin-1 (RIPK1 inhibitor), Necrosulfonamide (MLKL inhibitor) and Ferrostatin-1 (ferroptosis inhibitor) on TPH104c and TPH104m induced cell death. Morphological images of BT-20 cells pre-treated with **(a)** 50 µM of Necrostatin-1 **(b)** 1 µM of Necrosulfonamide (NSA) **(c)** 2 µM of Ferrostatin-1 and treated with 1 µM of TPH104c and TPH104m captured at 72 hours. Scale bar, 100 µm. The respective cell viability curves were obtained using IncuCyte S3 software based on phase-contrast images of BT-20 cells. The data represent the mean ± SD of three independent experiments performed in triplicate.

Table S1. The hydrogen bond occupancy table

**TPH104c**		
**Donor**	**Acceptor**	**Occupancy**
**LIG734-Side**	ASP146-Side	59.33%
**LYS38-Side**	LIG734-Side	30.79%
**LIG734-Side**	THR59-Main	0.60%
**SER39-Side**	LIG734-Side	1.20%
**GLN34-Side**	LIG734-Side	1.27%
**GLY149-Main**	LIG734-Side	0.82%
**SER35-Main**	LIG734-Side	1.20%

**TPH104m**		
**Donor**	**Acceptor**	**Occupancy**
**LIG734-Side**	ASP146-Side	35.73%
**LYS38-Side**	LIG734-Side	25.17%
**GLY149-Main**	LIG734-Side	7.34%
**GLY37-Main**	LIG734-Side	1.35%
**LIG734-Side**	GLN34-Side	0.37%
**SER35-Main**	LIG734-Side	3.60%
**GLN34-Side**	LIG734-Side	1.27%
**LIG734-Side**	LIG734-Side	0.22%
